# New infusion device for use in acquisition of images during endovascular procedures: an experimental model

**DOI:** 10.1590/1677-5449.200191

**Published:** 2021-06-11

**Authors:** Bruno Pagnin Schmid, Isadora Cardoso de Alencar, Carolina Masson, Giovani José Dal Poggetto Molinari, Fábio Hüsemann Menezes

**Affiliations:** 1 Universidade Estadual de Campinas – UNICAMP, Faculdade de Ciências Médicas, Campinas, SP, Brasil.

**Keywords:** angiography, endovascular procedures, aortic aneurysm, interventional radiology, angiografia, procedimentos endovasculares, aneurisma aórtico, radiologia intervencionista

## Abstract

**Background:**

The contrast power injector (CPI) is the gold standard method for injecting contrast with the pressure and flow needed to generate a satisfactory images during endovascular procedures, but it is an expensive tool, narrowing its wide-scale applications. One alternative is the manual injection (MI) method, but this does not generate the pressure required for adequate visualization of anatomy. It is therefore imperative to create an alternative low-cost method that is capable of producing high quality images.

**Objectives:**

To compare the injection parameters of a new mechanical device (Hand-Crank) created in a university hospital with the MI method and with the contrast power injector’s ideal values.

**Methods:**

A circulation phantom was constructed to simulate the pressure in the aorto-iliac territory and the injection parameters of the two methods were compared in a laboratory setting. Student’s *t* test and the Mann-Whitney test were used for statistical analysis. Three vascular surgery residents (the authors) performed the injections (each performed 9 tests using conventional manual injection and 9 tests using the Hand-Crank, totaling 54 injections).

**Results:**

There were statistical differences between the two methods (p<0.05) in total volume injected until maximum pressure was attained, pressure variation, maximum pressure, total injection time, and time to reach the maximum pressure.

**Conclusions:**

The Hand-Crank can achieve higher maximum pressure, higher average flow, and lower injection time than the manual method. It is a simple, low-cost, and effective tool for enhancing injection parameters in an experimental setup. It could help to produce higher quality images in a clinical scenario.

## Resumo

**Contexto:**

A bomba injetora é o método padrão-ouro para a injeção de contraste em aortografias. Entretanto, é uma ferramenta de alto custo, o que limita o seu uso. A injeção manual surge como alternativa, mas a pressão gerada com esse método é baixa, e, por isso, a qualidade das imagens não é usualmente satisfatória. Assim, a criação de um método de baixo custo capaz de gerar imagens de qualidade é imperativo.

**Objetivos:**

Comparar os parâmetros de injeção de um novo dispositivo mecânico (manivela articulada) criado em um hospital universitário com os parâmetros da injeção manual e com os valores ideais da bomba injetora.

**Métodos:**

Um simulador do território aórtico foi construído, e parâmetros de injeção entre os diferentes métodos em um cenário laboratorial controlado foram analisados. O teste *t* de Student e o teste de Mann-Whitney foram usados para análise estatística. Três residentes de Cirurgia Vascular realizaram os testes (nove usando o novo dispositivo, e nove usando a injeção manual, totalizando 54 injeções).

**Resultados:**

Houve diferença estatisticamente significativa (p < 0,05) entre os dois métodos, considerando os parâmetros: variação de pressão, pressão máxima, tempo de injeção, tempo até a pressão máxima e volume até a pressão máxima.

**Conclusões:**

A manivela articulada atingiu níveis superiores de pressão e de velocidade de injeção, com menor tempo de injeção do que a injeção manual. É um dispositivo simples, de baixo custo e com resultados comparáveis à bomba injetora, o que sugere seu uso potencial na geração de imagens satisfatórias em aortografias.

## INTRODUCTION

Precise deployment of endografts during endovascular aneurysm repair requires precise images of the landing zone showing anatomical features such as visceral aortic branches, proximal and distal aortic necks, and morphological characteristics of aneurysms.^1^^-^ 4 The contrast power injector (CPI) is the gold standard method for injecting contrast with the pressure and flow needed to produce satisfactory images.^5^ However, when the procedure is performed outside of the interventional radiology suite, generally in the regular operating room of a community hospital, a contrast power injector is usually not available. The CPI is also an expensive tool, which narrows its wide-scale applications, especially in developing countries. The manual injection (MI) method is an alternative option. However, MI may not generate the pressure and flow needed for adequate visualization of anatomy. It also involves risk of hand injury and fatigue due to the force applied to the syringe. In view of this situation, development of an alternative method capable of creating high quality images and less expensive than the contrast power injector is desirable. The aim of this study is to present the results of laboratory tests of a new mechanical device, that we have named the Hand-Crank (HC), designed to facilitate contrast injection during angiograms in a simple, low-cost, and reproducible manner. The HC was compared with the standard manual method for simulating the contrast power injector’s ideal values.

## METHODS

### The circulation phantom

**Figure 1:**
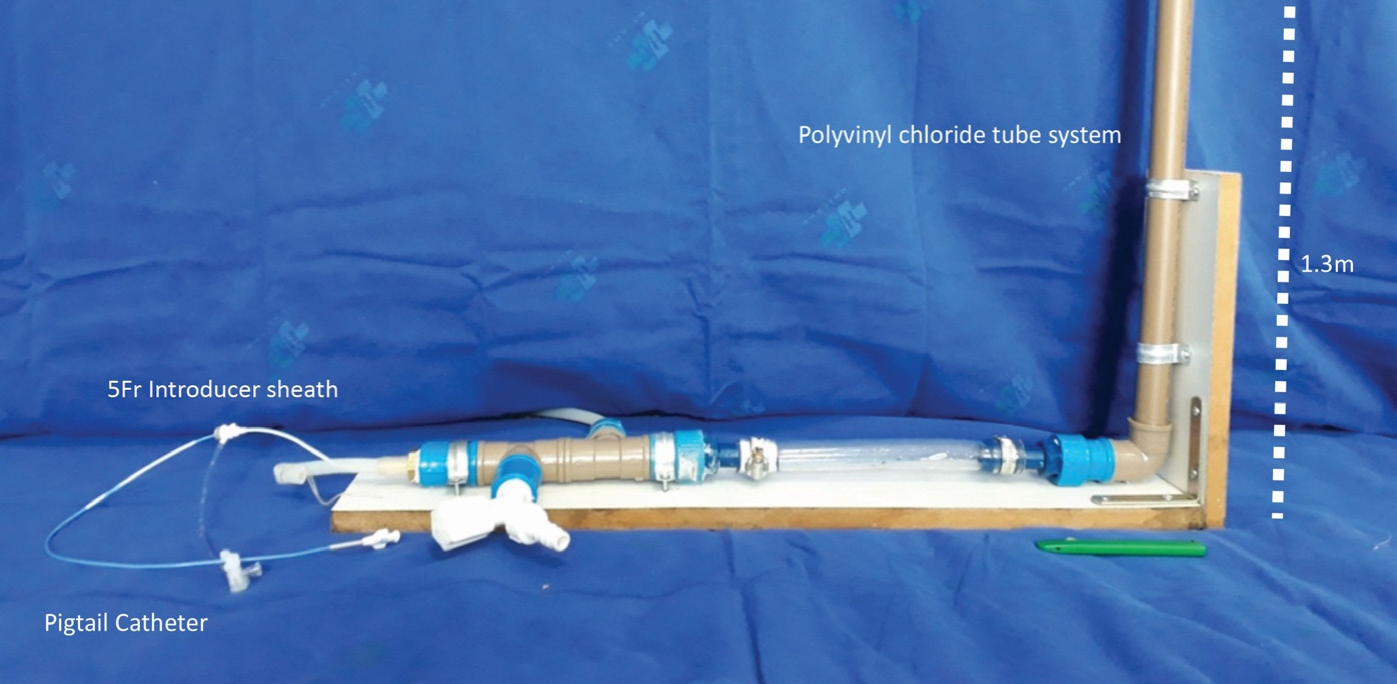
The circulation phantom constructed to simulate the aorto-iliac territory. The phantom is made from water pipes and connections with a vertical polyvinyl chloride pipe filled with a 1.3m water column to simulate the abdominal aorta.

A flow phantom simulating physiological arterial circulation parameters was used. It was constructed with components from a water pipe system to simulate the abdominal aorta. (Figure 1).

The pressure in the phantom was set to the adult physiological systolic arterial blood pressure of 120 mmHg in the aorto-iliac territory. To achieve this, the system was constructed incorporating a polyvinyl chloride pipe (Tigre S.A ®; Joinville, Brazil) filled with a water column measuring 1.3m in height along its vertical axis.

We used a 100cm pigtail catheter (Merit Medical®; South Jordan, UT) passed through a 5F introducer sheath (Medtronic®; Minneapolis, MN) to perform the tests. The water was changed completely after each injection.

### The Hand Crank

**Figure 2:**
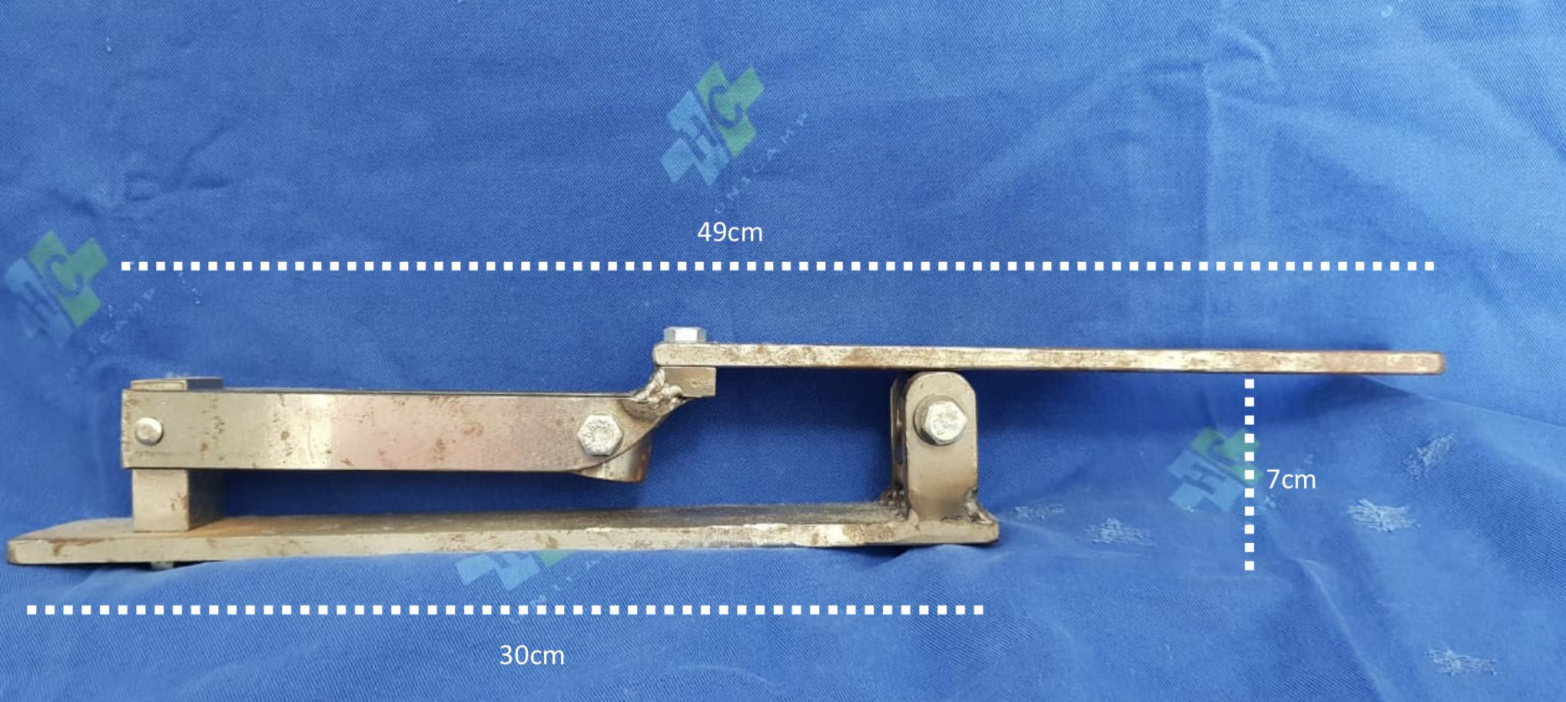
The Hand-Crank. A simple iron and steel portable tool.

**Figure 3:**
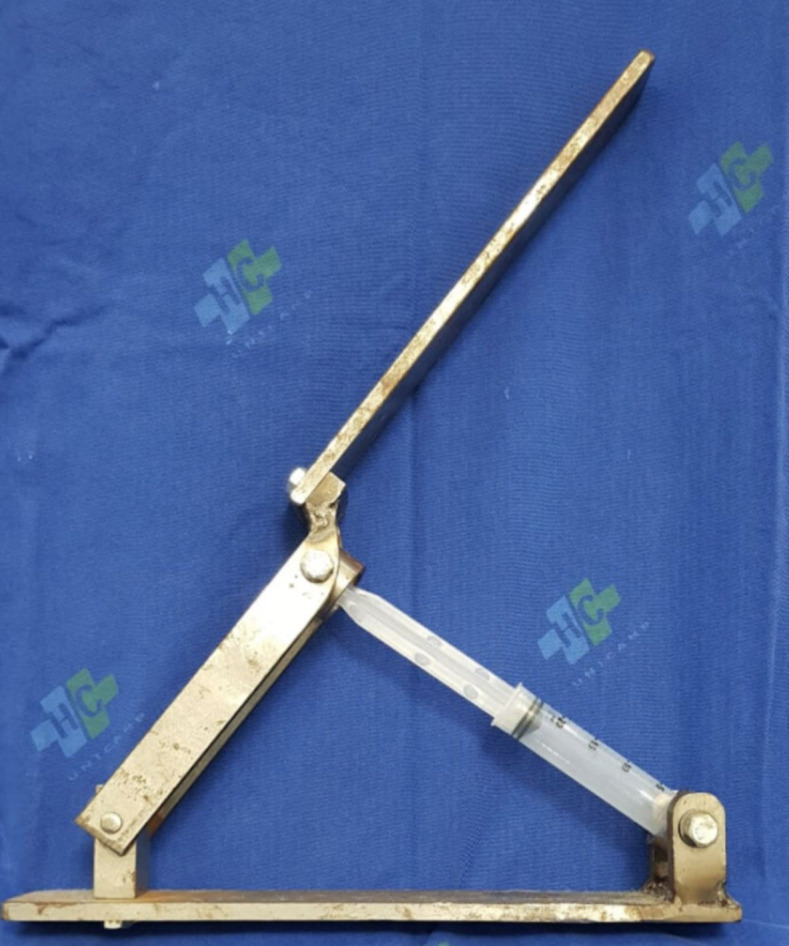
The Hand-Crank with a 20mL syringe attached. The articulated arm multiplies the pressure applied to the syringe.

The HC is a homemade 49x30x7cm iron and steel device that can be attached to a 20mL syringe (BD Plastipak®; Curitiba, Brazil). It is a portable tool that is suitable for any operating room, designed to facilitate its potential clinical use in the future. It was designed with an articulated arm that multiplies the pressure applied to the syringe (Figures 2 and 3).

### The tests

Tests were conducted to compare injection of a nonionic low osmolar contrast agent routinely used by our service (300mg/mL Iohexol GE Healthcare®; Shanghai, China) using the Hand-Crank and the standard manual injection method.

All tests were performed in an experimental surgery laboratory at a University Hospital in Brazil from January 2019 to March 2019. Three vascular surgery residents (co-authors) with appropriate expertise in use of syringes and catheters performed the injections. Each was instructed to inject 10mL of the contrast agent as fast as possible, simulating the pressure that they are used to applying during aortic interventions. Additionally, 2 pre-test injections (one using the manual injection and one using the Hand-Crank) were performed to enable the residents to familiarize themselves with the experimental setup. Finally, each resident performed nine consecutive injections using the conventional manual injection and nine using the Hand-Crank, totaling 54 injections for final analysis.

**Figure 4:**
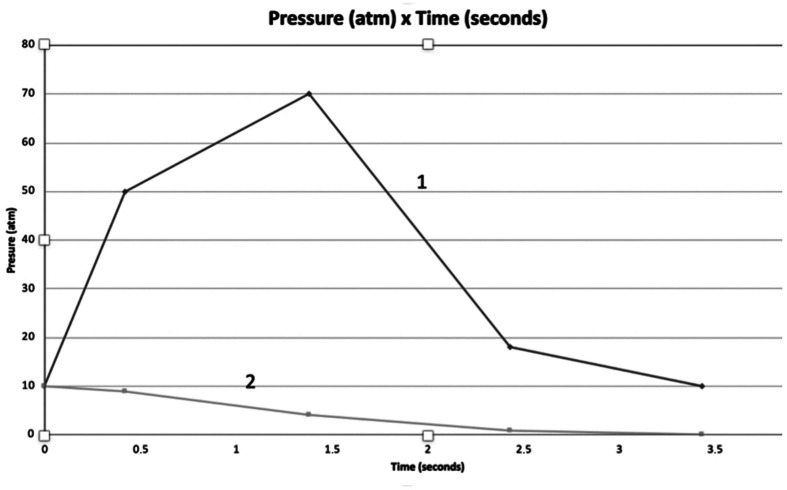
Average Pressure (atm) x Time (seconds) curve using the Hand-Crank (1) and manual injection (2). The Hand-Crank clearly generates higher pressure than the manual injection.

During the experiments, the 20mL syringe filled with the nonionic contrast agent was attached in series via a manometer (Boston Scientific®, Marlborough, MA) to the 5F x 100cm pigtail catheter (Merit Medical®., South Jordan, UT). Values for the pressure (atm) x time (seconds) were recorded on a microcomputer, generating analytical graphs from the data, as follows: total volume injected before the maximum pressure is reached, (Figure 4); maximum pressure; total injection time; time to reach the maximum pressure; average contrast flow; and average contrast flow until maximum pressure. The data obtained underwent statistical analysis using Student’s *t* test and the Mann-Whitney test to compare HC injections to manual injections.

Since this study was a pure benchwork experiment and did not involve any animal or human experimentation, it was not submitted for ethical review by the University Ethics Committee.

## RESULTS

**Figure 5:**
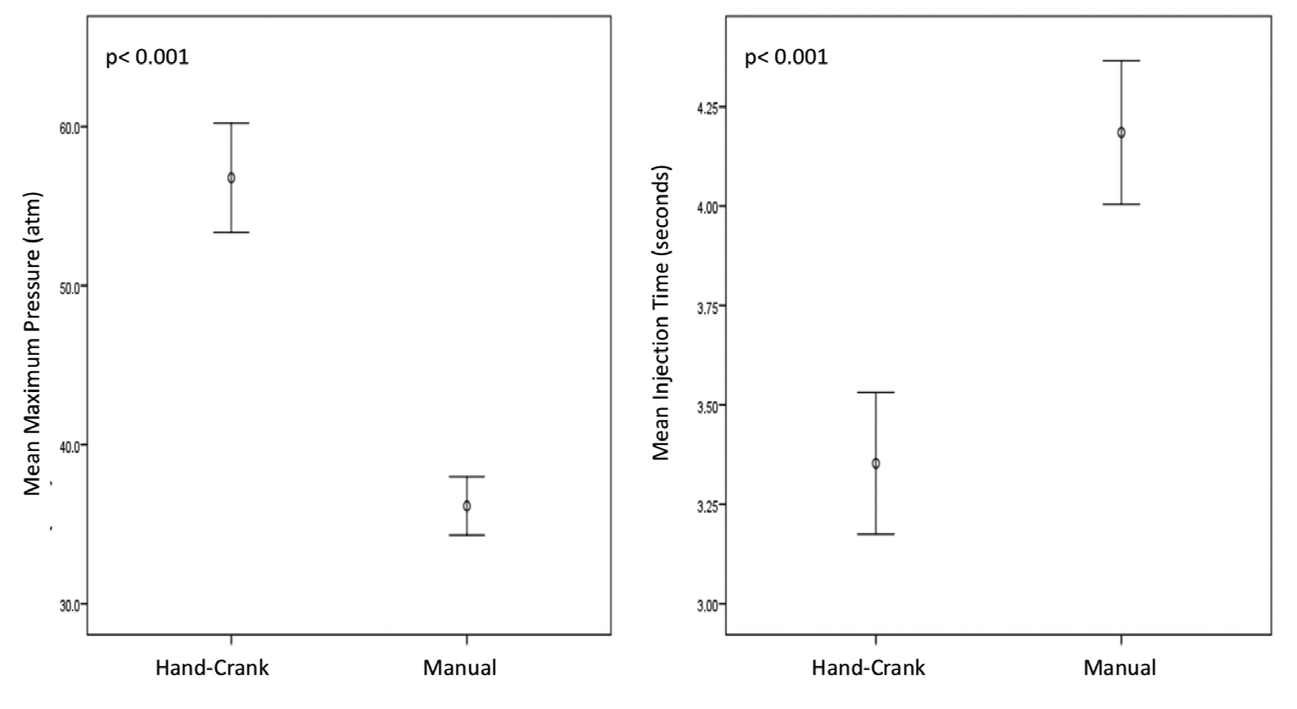
Comparison between the Hand-Crank and manual injection for the parameters “maximum pressure” and “mean injection time”.

**Figure 6:**
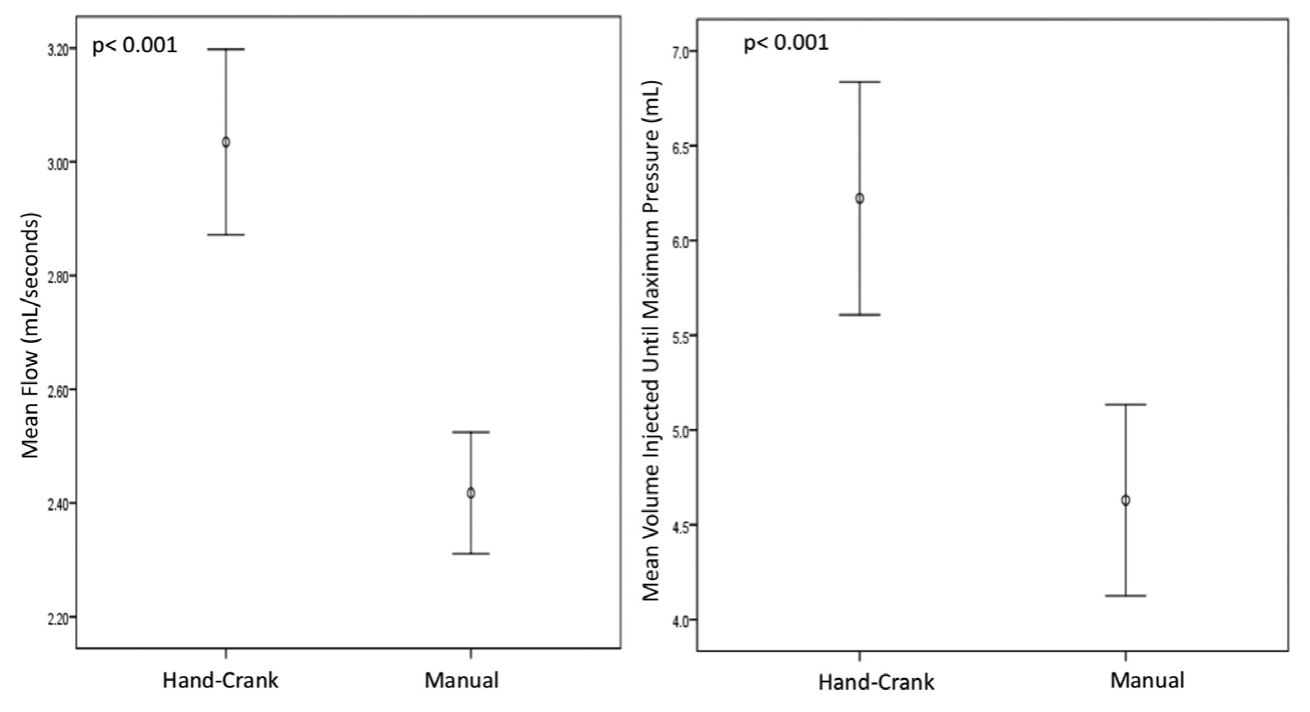
Comparison between the Hand-Crank and manual injection for the parameters “mean flow” and “mean volume injected until maximum pressure”.

Results for the comparison between HC and manual injections are presented in Tables 1 and 2 and in Figures 5 and 6.

**Table 1:** Comparisons between the Hand-Crank and manual injection for the following parameters: Volume injected until maximum pressure attained; pressure variation during the ascending phase (ΔP/ΔT ascending); pressure variation during the descending phase (ΔP/ΔT descending); and maximum pressure.

Parameters	Hand-Crank	Manual	p
Volume until MP (mL)			
Mean (SD)	6.22 (1.55)	4.63 (1.28)	< 0.001
Median (Range)	6 (4-9)	5 (2-7)	
ΔP/ΔT Ascending			
Mean (SD)	25.54 (10.13)	17.61 (11.2)	0.009
Median (Range)	23.36(10.67-54.72)	14.08 (7.24-60)	
ΔP/ΔT Descending			
Mean (SD)	37.72 (23.07)	14.16 (6.07)	< 0.001
Median (Range)	29 (17.59-131.58)	11.11 (7.56-30.93)	
MP (atm)			
Mean (SD)	56.78 (8.67)	36.15 (4.64)	< 0.001
Median (Range)	60 (40-70)	38 (30-46)	

atm= atmosphere; mL= milliliters; MP= maximum pressure; SD= Standard Deviation.

**Table 2:** Comparison between the Hand-Crank and manual injection for the following parameters: total injection time; time until maximum pressure attained; average flow; average flow until maximum pressure attained.

Parameters	Hand-Crank	Manual	p
Injection Time (s)			
Mean (SD)	3.35 (0.45)	4.19 (0.46)	< 0.001
Median (Range)	3.33 (2.48-4.56)	4.14 (3.35-4.9)	
Time to MP (s)			
Mean (SD)	2.01 (0.58)	1.79 (0.62)	0.172
Median (Range)	1.91 (1.06-3.52)	1.7 (0.5-3.1)	
Flow (mL/s)			
Mean (SD)	3.03 (0.41)	2.42 (0.27)	< 0.001
Median (Range)	3 (2.19-4.03)	2.42 (2.04-2.99)	
Flow until MP (mL/s)			
Mean (SD)	3.17 (0.58)	2.71 (0.56)	0.005
Median (Range)	3.16 (2.34-4.46)	2.51 (1.84-4)	

mL= milliliters; mL/s= milliliters per second; MP= maximum pressure; SD= Standard Deviation; s=seconds.

The Hand-Crank was capable of generating higher pressure values, higher injection flow, and shorter injection time than the manual method. We did not observe any hand injuries during our tests.

We also compared the injection time of the two methods with the gold standard values recommended by Schneider et al.^5^ (supposing a mean estimated volume of 7mL). Both methods performed poorly when compared to the contrast power injector’s ideal values (Table 3).

**Table 3:** Comparison between injection times using the Hand-Crank and the manual method and ideal values obtained using the contrast power injector: contrast administration volume (18-24mL); contrast administration time (3 seconds); contrast administration rate (6-8mL/second).^5^

Method	Mean (SD)	P	Mean Difference	CI
Manual	4.19 (0.46)	< 0.001	– 2.81	(– 3.00; – 2.63)
Hand-Crank	3.35 (0.45)	< 0.001	– 3.65	(– 3.83; – 3.47)

SD= Standard Deviation; CI= Confidence Interval.

## DISCUSSION

The concept of using pressure injection into vascular channels is governed by Poiseuille's law, according to which a laminar flow through a cylindrical pipe varies inversely to the viscosity of the medium and the length of the tube and directly to the pressure difference across the tube and the fourth power of the radius of the tube.^6^

From this rationale, angiographic image quality is strongly associated with injection of a contrast agent at the correct pressure. The pressure levels in the aortoiliac territory are especially high and difficult to attain with manual injection.^6^^,^ 7

According to Pasternak and Williamson, image quality is highly dependent on catheter diameter, injection flow, and administration route.^8^ In addition to these hemodynamic parameters, conditions related to the contrast agent’s properties such as osmolarity, viscosity, density, dose, and temperature (all of which remained constant for both methods analyzed) also play a key role during endovascular procedures.^9^

The gold standard values for contrast injection in the aorto-iliac territory (as recommended by Schneider et al.^5^ and obtained with the contrast power injector) are as follows: contrast administration volume (18-24mL), contrast administration time (3 seconds), contrast administration rate (6-8mL/seconds).^9^

In our tests, the manual method performed poorly with results far from these ideal values. These findings suggest that contrast injection without a specific pump system may be an inadequate option, compromising precise endograft deployment. Moreover, images acquired with manual injection are highly user‐dependent, since they are reliant on hand strength to generate enough contrast flow.

However, the new device was able to generate higher pressure values, higher injection flow, and shorter injection time than the manual method, with parameters similar to the contrast power injector. These promising results suggest that better images would be acquired during angiograms using the HC when compared to the MI. We did not observe any hand injuries during our tests, which suggests the HC is a safe device.

The HC is intended to be a low-cost and portable tool (49x30x7cm, 2.6Kg), suitable for any operating room and affordable by any hospital. Moreover, it can reduce the risk of hand injury due to syringe fracture while multiplying the pressure generated during aortic interventions, with the potential to facilitate production of better images without use of a power injector, enabling precise endograft deployment.

As tested, the HC is a prototype still in development and is not the final version. The authors intend to perform a future clinical study, in which the HC would be positioned on a sterile back-table and manipulated freely by the vascular surgeon during endovascular procedures. A stainless-steel version must be produced for this purpose that could be sterilized following the same protocols for any surgical instrument. A plastic 3D-printed version could also be considered. In case of multiple injections, simple substitution of the syringe can be performed easily. Furthermore, addition of a manometer could facilitate control of the desired pressure during the procedure and during training in how to handle the prototype.

This study’s limitations include its small sample size and the experimental setup which was focused only on the characteristics of the injection pressure and time. To further explore the concept presented would require a clinical phase in a randomized clinical trial to confirm its potential use compared to the contrast power injector, taking into consideration other determinant factors that influence image quality, such as patients’ hemodynamic status, contrast material used, image noise, and technical radiological parameters.^8^ Other elements such as injection material costs and procedure duration must also be analyzed. Additionally, the tests were performed by vascular surgery residents intimately involved in creation of the device and, therefore, with considerable experience with its use. Since this is an innovative device, a practical training period may be required for other users.

Finally, the authors recognize that inclusion of a CPI group would have yielded important information for this study, since this device is the gold-standard method for contrast injection. However, the primary objective was the comparison between the new device and manual injection, targeting hospitals that cannot afford a CPI. Furthermore, inclusion of the CPI would have involved additional cost that was not accounted for in this project. We did compare our results to the ideal values achieved with the CPI for the purposes of discussion of the HC’s potential applications.

## CONCLUSIONS

The Hand-Crank proposed in this project is an efficient device for increasing pressure and flow of contrast injections during angiograms. It generates higher pressure and flow levels than the manual injection, reaching values that can be comparable with the contrast power injector. Therefore, the HC is a tool with potential for acquisition of better images in endovascular procedures. It is a simple, low-cost device and is therefore affordable for many additional hospitals.
